# Plant-made pharmaceuticals

**DOI:** 10.5511/plantbiotechnology.24.0716a

**Published:** 2024-09-25

**Authors:** Noriho Fukuzawa, Kouki Matsuo, Go Atsumi, Yasushi Tasaka, Nobutaka Mitsuda

**Affiliations:** 1 Bioproduction Research Institute, National Institute of Advanced Industrial Science and Technology (AIST), 2-17-2-1 Tsukisamu-Higashi, Toyohira-ku, Sapporo, Hokkaido 062-8517, Japan

**Keywords:** plant molecular farming, plant-made pharmaceutical

## Abstract

Plant-made pharmaceuticals (PMP) have great potential in terms of production costs, scalability, safety, environmental protection, and consumer acceptability. The first PMP were antibodies and antigens produced in stably transformed transgenic plants in the around 90s. Even though the effort using stable transgenic plants is still going on, the mainstream of PMP production has shifted to transient expression in *Nicotiana benthamiana*. This system involves the expression vectors by *Agrobacterium*, and its efficiency has been improved by the development of new vector systems and host engineering. The COVID-19 outbreak accelerated this trend through efforts to produce vaccines in plants. Transient expression systems have been improved and diversified by the development of plant virus vectors, which can be classified as full and deconstructed vectors. Full virus vectors spread systemically, allowing for protein production in the entire plant. Compared with conventional agroinfiltration vectors, excellent virus vectors result in higher protein production. Engineering of host plants has included knocking out gene-silencing systems to increase protein production, and the introduction of glycan modification enzymes so that plant-made proteins more resemble animal-made proteins. Hydroponic cultivation systems in plant factories and environmental controls have contributed to efficient protein production in plants. Considering their advantages and small environmental impact, PMP should be more widely adopted for pharmaceuticals’ production. However, the initial investment and running costs of plant factories are higher than open filed cultivation. The next objectives are to develop next-generation low-cost plant factories that use renewable energy and recycle materials based on the idea of circular economy.

## Introduction

In recent years, demand for a shift toward bio-manufacturing that reduces the burden on the global environment has been increasing. Plants are competent producers in an ecosystem, capable of fixing carbon dioxide and producing organic matter. Research and development on the use of plants as expression platforms and biotechnological strategies to produce high value-added substances that are useful to humans is called “Plant molecular farming (PMF)”. It began in the late 1980s and has continued to grow steadily for over 30 years (Schillberg and Finnern 2021). The final products of PMF include proteins, chemicals, pharmaceuticals, cosmetics, reagents, and industrial enzymes. A body of scientific knowledge has accumulated on the production of chemicals and foreign proteins that are not innately produced by plants.

While most industrial recombinant protein production is performed using cultures of yeasts, mammalian cells, and bacteria such as *Escherichia coli* (Walsh and Walsh 2022), only a small number of approved biopharmaceuticals are produced using insect cells, transgenic animals, and transgenic chicken platforms. These recombinant proteins include biopharmaceuticals with high market value and a rapidly growing demand. Among the final products of PMF, high value-added proteins such as biopharmaceutical raw materials, as well as diagnostic and therapeutic proteins for humans and animals, are collectively referred to as “Plant-made pharmaceuticals” (PMP). These products include recombinant proteins similar to those produced using other cell-based manufacturing processes, including monoclonal antibodies, vaccines, enzymes, receptor modulators (e.g., hormones, cytokines, cell growth factors), extracellular matrices, and blood components. Plant-based manufacturing has been described as a “promising” or “emerging” platform, rather than a replacement for traditional manufacturing processes, and has been used as a development platform for the production of PMP to treat orphan diseases, locally endemic diseases, and other diseases with a low prevalence (Lobato Gómez et al. 2021). The PMP research and development field involves the development of vaccines and medicines not only for human use but also to prevent or reduce the occurrence of highly contagious and economically important diseases in livestock (Su et al. 2023) and farmed fish (Fukuzawa et al. 2010b). Research is also progressing to produce virus nanoparticles derived from plant viruses in plants for use as nanocarriers in the medical field, with applications as self-adjuvants and drug-delivery systems (Chung et al. 2022).

In this review, we focus on the production of valuable pharmaceutical proteins and review the global development status of PMP manufacturing and the development of fundamental technologies for social implementation.

## Manufacturing advantages of PMP

Plant expression platforms offer several advantages over other expression systems (Matsumura 2012). First, they are considered safe because of the low risk of contamination with zoonotic pathogens and endotoxins, which can occur with conventional culture processes. Second, they are cost-effective and scalable (if they are cultivated in an open field), making them an inexpensive production system (Donini and Marusic 2019). Adjusting the scale of production to meet demand is relatively easy, and the initial cost of capital investment is low. Studies have reported that the cost of plant expression platforms is lower than that of other production systems in terms of socio-economic potential and commercial feasibility (Nandi et al. 2016; Tusé et al. 2014). Third, plants are edible. The ability to orally ingest edible parts such as seeds and fruits is a unique advantage of producing useful substances using genetically modified (GM) plants.

Plants are higher eukaryotes and have intracellular secretory pathways similar to those of mammals. Therefore, by using a reliable expression system based on transgenic plants with the gene of interest (GOI) stably integrated into the nuclear genome, or plants transiently expressing the GOI, it is possible to produce proteins with higher-order structures because plants have the cellular machinery to carry out eukaryotic post-translational modifications. Chloroplasts, which are plant-specific organelles, can also host foreign genes in their genome, and can stably and abundantly express recombinant proteins (Zhang et al. 2017). The genetic characteristics of chloroplasts are similar to those of prokaryotes, and therefore recombinant proteins that do not require glycan modifications can be produced in them similarly to *E. coli* platforms. Plant expression platforms also allow the use of plant pathogens such as plant viruses (Gleba et al. 2007). These are used as expression vectors to produce recombinant proteins through transient expression. Plant viruses are particularly safe for use in this context because they cannot infect humans. By using the native properties of plant viruses, namely their ability to infect and replicate within host plants, it is possible to transiently produce a large amount of recombinant proteins within a few days. Because of their operability and high expression levels, transient expression systems are currently the mainstream method of recombinant protein production using plants. Plant cell suspension cultures have also been employed as protein production platforms (Schillberg et al. 2013). Although it is relatively easy to collect and purify the target protein, especially if it is produced as a secreted protein, in general, the initial investment cost of plant cell suspension cultures are higher than those of the other plant expression methods described above (Donini and Marusic 2019; Venkataraman et al. 2023). It can be used as a bridging technology between plant expression systems and other expression platforms because it is compatible with the cell-based manufacturing infrastructure that is currently used in industrial production.

## Manufacturing history and current trends of PMP

Although the scientific, economic, and social advantages of the plant-based manufacturing platform are well recognized, it has been pointed out that “PMP has always faced a double jeopardy of the risk of a new product and new platform” (Benvenuto et al. 2023). PMP production seems to have had its ups and downs, unable to break into an industrial structure usually employing cell-based manufacturing processes. However, the production of pharmaceuticals using methods that reduce the burden on the global environment is an urgent issue, and there is a possibility that social demand will suddenly increase, as in the case of the development of mRNA vaccines during the COVID-19 pandemic, which could make PMP advantageous. This chapter reviews the research and development that has been an important landmark in the development of PMP with each expression system to date (summarized in Table 1).

**Table 1:** Table 1. Major landmarks of PMP.

Year	Manufacture	Product	Purpose	Host	Type	References
1992	AgriStar Inc.	Hepatitis B virus surface antigen (HBsAg)	Human vaccine	*N. tabacum*	Transgenic plant	Mason et al. 1992
1996	Texas A & M University	Norovirus genotype 1 capsid protein	Human edible vaccine	Potato	Transgenic plant	Mason et al. 1996
1998	Boyce Thompson Institute for Plant Research	*E. coli* heat-labile enterotoxin B subunit	Human edible vaccine	Potato	Transgenic plant	Mason et al. 1998
2003	Boyce Thompson Institute for Plant Research	Hepatitis B virus surface antigen (HBsAg)	Human edible vaccine	Potato	Transgenic plant	Smith et al. 2003
2006	Dow AgroSciences LLC	Poultry vaccine against Newcastle disease virus	Poultry vaccine	*N. tabacum* NT-1 suspension culture cells	Transgenic culture cell	Vermij and Waltz 2006
2012	Protalix BioTherapeutics Inc.	Human therapeutic product for Gaucher’s disease	Human medicine	Carrot suspension culture cell	Transgenic culture cell	Shaaltiel et al. 2007
2012	ORF genetics Inc.	Human epidermal growth factor	Human cosmetics	Barley	Transgenic plant	Schouest et al. 2012; Magnusdottir et al. 2013
2013	AIST; Hokusan Inc.	Canine interferon-α	Pet medicine	Strawberry	Transgenic plant	Matsumura and Tabayashi 2022
2014	Bayer Innovation GmbH	Personalized vaccine for follicular Non-Hodgkin’s lymphoma	Human medicine	*N. benthamiana*	Transient expression	Bendandi et al. 2010; Tusé et al. 2015
2014	Mapp Biopharmaceutical Inc.	Neutralizing antibodies for Ebola virus disease	Human medicine	*N. benthamiana*	Transient expression	Olinger et al. 2012; Pettitt et al. 2013; Qiu et al. 2014
2015	St. George’s University of London; Fraunhofer IME; RWTH Aachen University	HIV neutralizing antibody 2G12	Human medicine	*N. tabacum*	Transgenic plant	Ma et al. 2015
2019	BioApplications Inc.	Swine fever virus E2 protein	Poultry vaccine	*N. benthamiana*	Transgenic plant	Park et al. 2019
2022	Medicago Inc.	COVID-19 vaccine	Human vaccine	*N. benthamiana*	Transient expression	Ward et al. 2021

### 1) Transgenic plant cell suspension culture

A PMP produced using GM plant cells grown with aseptic culture methods was the first to receive regulatory approval. In 2006, Dow AgroSciences LLC developed a subunit vaccine, “Concert”, to protect poultry against Newcastle disease virus, and the United States Department of Agriculture approved it as the first PMP (Vermij and Waltz 2006). The vaccine antigen protein, hemagglutinin-neuraminidase glycoprotein, was produced by suspension cells of transgenic *Nicotiana tabacum* NT-1 stably expressing the encoding gene. The vaccine product was then manufactured by extraction and purification from the cultured cells, but it was not commercialized because of strategic business decisions (Fox 2006). In 2012, the US Food and Drug Administration (FDA) approved a human therapeutic product developed by Protalix BioTherapeutics Inc. for enzyme replacement therapy to treat Gaucher’s disease. The product compensates decreased β-glucocerebrosidase activity in affected patients. The enzyme “Elelyso” (Shaaltiel et al. 2007), a recombinant human β-glucocerebrosidase, was produced by suspension-cultured carrot cells expressing its encoding gene, and it was purified and licensed for injection by Pfizer. Thus, the first PMP preparation for human use was produced using a plant cell culture platform.

### 2) Transgenic plant-based manufacturing

In 1992, tobacco (*N. tabacum* cv. Samsun) was used to express the hepatitis B virus surface antigen (HBsAg), resulting in the formation of virus-like particles with the same properties as those of HBsAg produced in yeast (Mason et al. 1992). This was the first attempt to create a vaccine using GM plants. Subsequently, potato tubers were used to express HBsAg, the *E. coli* heat-labile enterotoxin B subunit, and the norovirus genotype 1 capsid protein (Mason et al. 1996, 1998; Smith et al. 2003). When these tissues were orally administered to animals, they induced the production of antigen-specific antibodies. On the basis of these data, the investigators filed Investigational New Drug applications and gained approval for three human clinical trials. In all cases, the volunteers ate raw peeled potato tubers, and as a result, antibodies were raised. This indicated that mucosal immunization was successful, and the trial was conducted up to phase I/II (Arntzen et al. 2005). Although it was stopped at this phase, this demonstrated the concept of the “edible vaccine”, which led to the development of PMP with stable expression systems (Arntzen 2015).

In the 2000s, stable expression systems using transgenic plants became mainstream and their use was promoted worldwide (Ma et al. 2003). In the United States, open-field cultivation and greenhouse cultivation with sunlight were common practices. However, in 2002, the European Medicines Agency and the US FDA each promulgated guidelines for the field cultivation of GM plants to produce human and animal pharmaceutical raw materials and industrial raw materials. Revised versions were issued in 2007 by the US Animal and Plant Health Inspection Service, and in 2008 by the European Medicines Agency. Although none of the guidelines prohibited cultivation in fields for non-food or non-feed purposes, they became stricter from the perspective of preventing the spread of introduced genes, and included guidelines for the education and training of workers (Matsumura 2012). However, when cultivating plants to produce PMP as raw materials for pharmaceuticals, it is important to consider the process of pollen dispersal from GM plants, to maintain stable production throughout the year, and to ensure a high level of cleanliness. In 2007, the world’s first pilot-scale cultivation facility adhering to these standards was established at the National Institute of Advanced Industrial Science and Technology (AIST) in Japan (https://www.aist.go.jp/Portals/0/resource_images/aist_j/aistinfo/aist_today/vol07_08/vol07_08_p14_p15.pdf (Accessed Sep 2, 2024)). This facility is completely enclosed, and the plants are grown with a hydroponic cultivation system under artificially controlled environmental conditions (Goto and Matsumura 2009; Takasuna et al. 2009). Many studies have been conducted using GM plants of various species expressing PMP in this closed-type GM plant factory (Matsumura and Goto 2009).

In 2013, the Japanese Ministry of Agriculture, Forestry and Fisheries approved a canine periodontal disease mitigant developed by AIST and Hokusan Co. Ltd. This product, which was the world's first edible-type PMP drug, was made from transgenic strawberry fruit expressing canine interferon-α (Matsumura and Tabayashi 2022). Cultivation of this strawberry line and manufacturing of formulations were carried out in the closed-type GM plant factory mentioned above. In 2023, Hokusan Co. Ltd. got the approval to extend the application to cats as well. The product, marketed as “InterBerry α”, is currently used in veterinary clinics in Japan (https://hokusan-kk.com/item/).

The Pharma-Planta project (2004–2011), which was carried out by 12 European and American countries and the Republic of South Africa, developed the *N. tabacum* L. Petit Havana cultivar SR1 expressing the human immunodeficiency virus (HIV)-neutralizing antibody 2G12, and conducted a phase I study (Ma et al. 2015). Good manufacturing practice (GMP)/good agricultural and collection practice-compliant upstream production of 2G12 was achieved using plants grown in a glass greenhouse (Sack et al. 2015).

In 2019, BioApplications Inc. received approval from the Animal and Plant Quarantine Agency under the Korean government for their injection-type subunit vaccine, HERBAVAC CSF Green Marker, which was designed to protect pigs from classical swine fever virus (https://www.bioapplications.global). This vaccine was produced by purifying the swine fever E2 protein from transgenic *N. benthamiana* plants cultivated in plant factory under artificially controlled environmental conditions (Park et al. 2019).

Although human epidermal growth factor is not a pharmaceutical, was produced in transgenic barley plants and the purified protein was used as a bioactive cosmetic ingredient. This protein was marketed by ORF Genetics (Magnusdottir et al. 2013; Schouest et al. 2012). Year-round production of this protein was achieved using plants growing in glass greenhouses built on an isolated plateau of basalt lava with no natural vegetation around, powered by geothermal energy.

### 3) Transient expression-based manufacturing

In 2009, the European Food Safety Association GM organism panel issued a scientific opinion about the risk assessment of GM plants. It was recommended that GM plants should be used for non-food or non-forage purposes (EFSA Panel on Genetically Modified Organisms 2009). Although a wide variety of edible plant species had been used during earlier periods of development, this opinion triggered PMP platforms in Europe and the United States to move towards transient expression systems using *N. benthamiana* because of its ease of cultivation and handling, and its ability to express proteins efficiently and at high levels (Matsumura 2012).

In 2014, Bayer Innovation GmbH completed a phase I clinical study on the safety and immunogenicity of personalized vaccines to treat patients with follicular Non-Hodgkin’s lymphoma (Bendandi et al. 2010; Tusé et al. 2015). The expression platform used was the MagnICON system developed by IconGenetics Inc. (Giritch et al. 2006; Marillonnet et al. 2004). This system is a rapid and highly productive tool using *N. benthamiana*. It has accelerated the development of transient expression systems for the production of recombinant proteins, and, in the development of this vaccine, it enabled the rapid production of a multitude of idiotypes derived from individual patients. Although a personalized immunogenic full-idiotype IgG vaccine could be produced within weeks after obtaining biopsies from Non-Hodgkin’s lymphoma patients and a phase I human clinical trial was successfully completed, further development was halted because of business decisions.

In 2014, U.S.-based Mapp Biopharmaceutical Inc. developed ZMapp, a cocktail of three neutralizing antibodies produced by plants to treat Ebola virus disease (Olinger et al. 2012; Pettitt et al. 2013; Qiu et al. 2014). The product was manufactured on contract by Kentucky BioProcessing Inc. using a transient expression system in *N. benthamiana* (Swope et al. 2021). During the Ebola virus disease epidemic in west Africa, ZMapp received a temporary emergent approval from the FDA for compassionate use and was administered to affected patients. Five out of seven ZMapp-treated patients survived (Meyers et al. 2015). It was the first time that therapeutic antibodies produced in *N. benthamiana* leaves had been administered to humans. This demonstrated the advantages of a plant-based transient expression system to respond to an emergency situation by quickly producing GMP-quality antibodies.

In 2022, a plant-made COVID-19 vaccine for use in humans was developed by Medicago/Tanabe-Mitsubishi Inc., and was approved under the Food and Drug regulations in Canada. It was licensed as “Covifenz” within 24 months of the initial vaccine design (https://www.canada.ca/en/health-canada/services/drugs-health-products/covid19-industry/drugs-vaccines-treatments/vaccines/medicago.html). The vaccine was expressed a using transient expression system in *N. benthamiana* leaves (Ward et al. 2021). Medicago announced plans to build a new production facility, spread over 9 ha, that would be capable of supplying up to one billion vaccine doses per year. Although this was the world’s first publicly approved plant-made injection-type human vaccine, the company ceased operations in February 2023 due to strategic economic reasons. In December 2023, Medicago’s research and development assets, intellectual property, and facilities were transferred to another Canadian company, Aramis Biotechnologies Inc. (https://aramisbiotechnologies.com). This allowed Aramis Biotechnologies to leverage the original investment from the Canadian government to Medicago, and meant that key domestic assets were retained in Canada (https://www.canada.ca/en/innovation-science-economic-development/news/2023/12/agreement-reached-on-retaining-medicagos-strategic-research-and-development-assets-in-canada-and-recovering-payment-from-medicago.html).

### 4) Current trends after the COVID-19 pandemic

**Figure 1:**
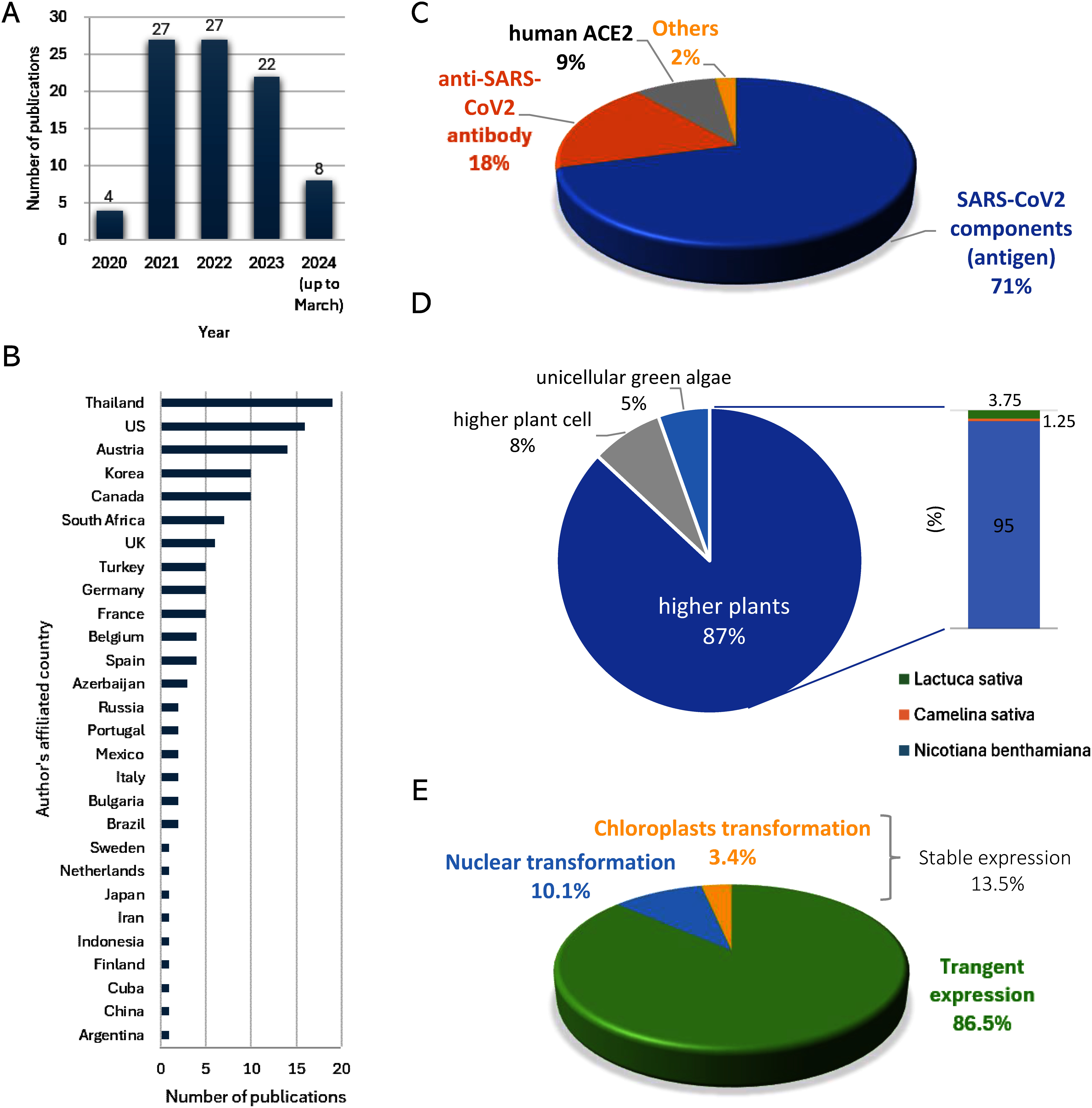
Figure 1. Summary of PMP research papers related to COVID-19/SARS-CoV2 (as of March 31, 2024). (A) Changes in the number of PMP research papers related to COVID-19/SARS-CoV2. (B) Countries that authors are affiliated with which target COVID-19/SARS-CoV2 in PMP research and the number of published papers. (C) Breakdown of COVID-19/SARS-CoV2 related substances targeted for plant expression. (D) Platform plants and plant species used for expression of COVID-19 related substances. “Higher plant cell” includes *Nicotiana tabacum* BY-2 cell and *Medicago truncatula* A17 cell. “Unicellular green algae” includes Chlorella and *Chlamydomonas reinhardtii*. (E) Plant expression systems used for expression of COVID-19 related substances.

During the COVID-19 pandemic, some low- and middle-income countries were unable to acquire a sufficient supply of imported COVID-19 vaccines. This prompted research and development in those countries to produce pharmaceuticals and other products using plant expression systems (Murad et al. 2020; Tsekoa et al. 2020). In 2022, Baiya Phytopharm Inc. in Thailand initiated a phase I clinical trial of a candidate COVID-19 vaccine produced by transient expression in *N. benthamiana* in a GMP facility. In the latest review, the Data Safety Monitoring Board identified an acceptable safety profile and recommended the trial to continue for the second cohort (https://baiyaphytopharm.com/announces-preliminary-results-of-the-phase-1/). In 2020, Cape Biologix Technologies, a subsidiary of Cape Bio Pharms Ltd. in South Africa, established a system to produce an anti SARS-CoV-2 N protein antibody for use as a research or diagnostic reagent by transient expression in *N. benthamiana*. This technology can be applied to produce therapeutic drugs in the future if manufactured under GMP compliant conditions (https://capebiologix.com). As of the end of 2023, 28 countries had reported research and development related to COVID-19 PMP (Figure 1 and Supplementary Table S1). The low initial cost of plant expression systems compared to the usual cell-based manufacturing and the need to produce medicines against endemic diseases in a limited locality have led to active participation in PMP development by countries without the infrastructure to produce such materials using conventional methods.

According to the report in April 2023 which took an impact of COVID-19 on the market into account, predicted that, between 2021 and 2031, the global market for plant-based biologics is expected to grow by 4.8% at a compound annual growth rate, reaching USD 182.9 million in 2031 (https://www.researchdive.com/150/plant-based-biologics-market). Although only a fraction of the PMP under development has been approved by regulatory authorities, the variety of plant species, modes of expression, and dosage forms highlight the diversity of plant expression systems, and the potential for plant expression technology to be a new industry in the future.

## Overview of currently used plant expression systems

**Figure 2:**
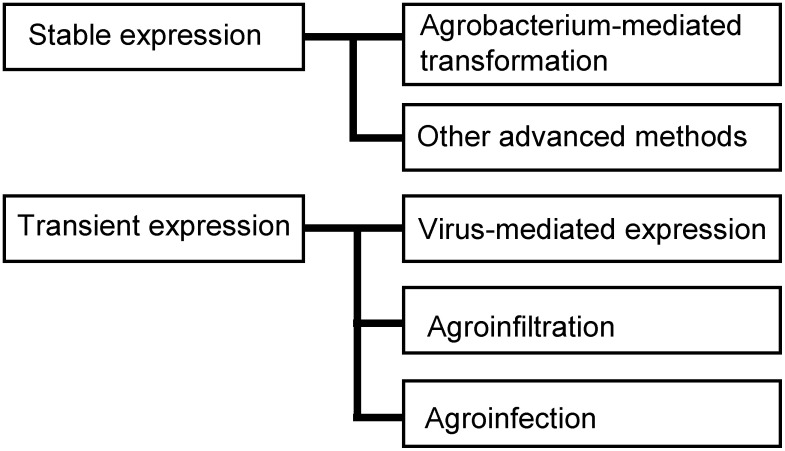
Figure 2. Overview of plant expression systems for PMP production.

The expression systems used in the development of PMF/PMP can be broadly divided into two main categories; stable expression systems and transient expression systems (Figure 2). For stable expression systems, transgenic plants, which are usually made by *Agrobacterium tumefaciens* (*Rhizobium radiobacter*)-mediated method, are mainly used, and although it takes a long time to produce them, it is possible to establish a fixed lineage. Transient expression systems use plant virus vectors, agroinfiltration with *A. tumefaciens*, or agroinfection that fuses these two methods. These methods use genes derived from plant pathogens and allow for high levels of protein expression in a short time. The agroinfiltration method, which is the most widely used, uses *A. tumefaciens*, a plant tumorigenic bacterium with a high capacity to transfer DNA to plants and finally insert the T-DNA region of a binary plasmid into the plant genome in a certain efficiency. An expression cassette containing a promoter for plant expression (e.g., the 35S promoter from cauliflower mosaic virus [CaMV]), a foreign GOI, and a terminator sequence is delivered into the plant cell via *Agrobacterium*. The target recombinant protein should be expressed in almost all leaves and therefore, the vacuum agroinfiltration method is used to inoculate vector-bearing *Agrobacterium* into the entire plant body.

The development of plant viral vector methods for recombinant protein production depends mainly on the characteristics of the original plant virus (genome sequence), and the host plant range of the original virus. Vector development has progressed using viruses with high multiplication rates in plants and/or a wide host range. The agroinfection method allows for high level of gene expression by combining the two previously described methods. In this system, a plant virus vector is inserted in the T-DNA region of the binary plasmid as a replicon that can self-replicate within plant cells. When *Agrobacterium* harboring the binary plasmid infects the plant, the plant virus vector with the GOI can self-replicate because it retains its own capacity for replication. The use of this type of vector has rapidly increased since it was first demonstrated that the GOI could be expressed through the agroinfection method using a CPMV vector with an inserted green fluorescent protein (GFP) gene (Liu and Lomonossoff 2002). This method does not necessarily rely on the systemic transfer capacity of the plant virus vector itself because the use of the vacuum agroinfiltration method ensures that the plant virus vector is introduced into the entire plant body. Large amounts of recombinant protein can be expressed in the plant body if the capsid/coat protein (CP) gene is deleted. Several second-generation virus vectors have been developed for use with the agroinfection method, including deconstructed viral vectors (see the next section for details) such as TMV and PVX-based magnICON vectors, the TMV RNA-based overexpression (TRBO) vector (Lindbo 2007), the CPMV-based pEAQ vector (Sainsbury et al. 2009), the Geminivirus bean yellow dwarf virus (BeYDY)-based deconstructed vector (Chen et al. 2013) and the cucumber mosaic virus (CMV)-based deconstructed vector (Fukuzawa et al. 2018).

## Plant virus vectors for transient recombinant protein expression

Many plant virus vectors have been used for transient expression of recombinant proteins. In this section, we introduce popular plant virus vector systems, but we do not describe the kinds of proteins that are actually produced using these vectors in detail (see reviews for details of the expressed proteins: Ibrahim et al. 2019; Lico et al. 2008). When a plant virus vector is delivered into a host plant cell, the virus replicates and moves to neighboring cells via the plasmodesmata (cell-to-cell movement). Eventually the virus enters the phloem cells, spreads to the distal tissues (systemic infection), and produces viral proteins in the infected systemic tissues. Plant virus vectors have been developed based on many plant viruses, including DNA and RNA viruses (Porta and Lomonossoff 2002), and can be classified as full or deconstructed vectors (Gleba et al. 2004). Full virus vectors are able to infect host plants systemically in an autonomous manner as observed in the original wild-type virus infection. Deconstructed vectors are replicons that cannot infect systemically because the part of the viral genome required for full infectivity has been deleted.

**Figure 3:**
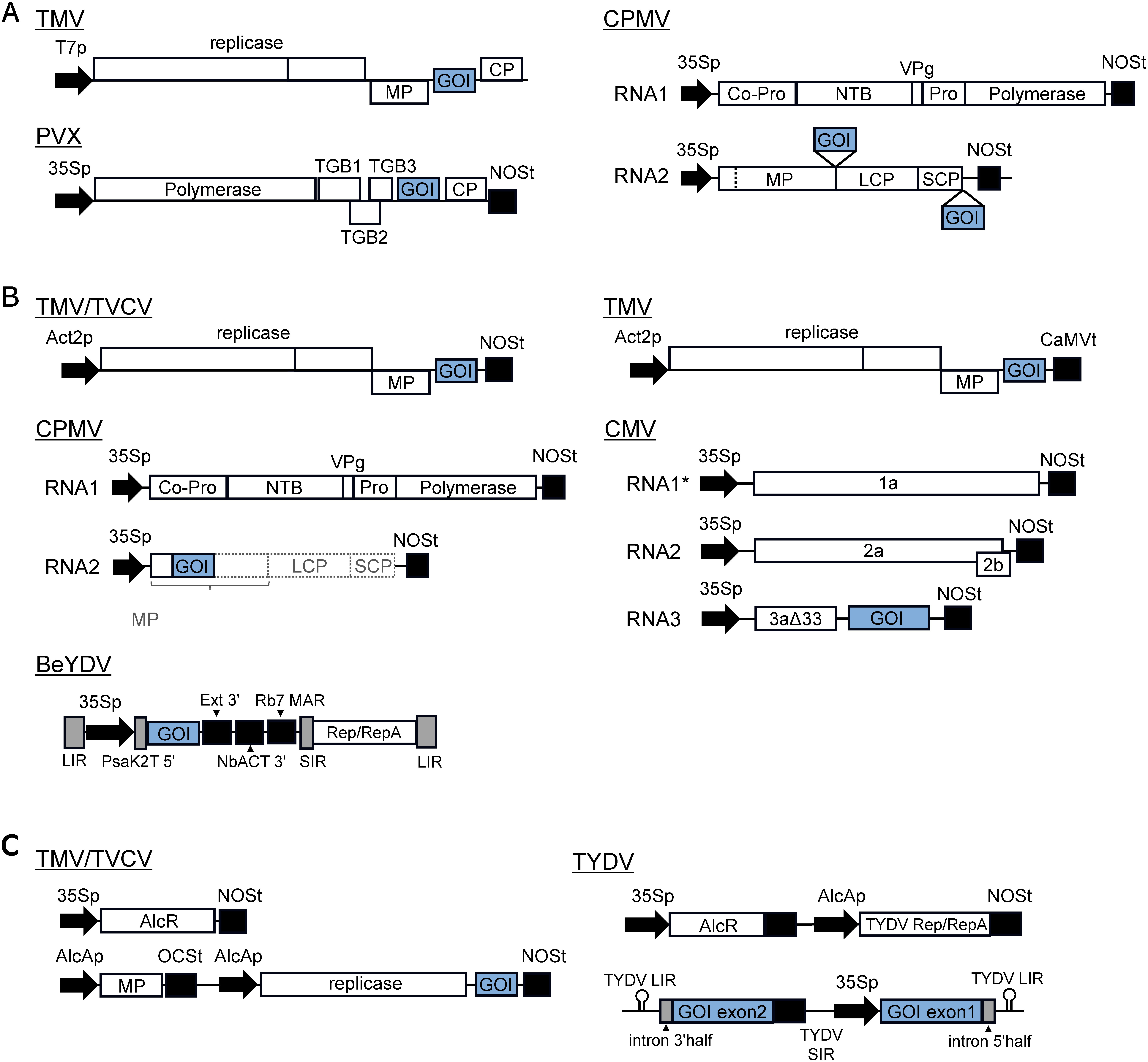
Figure 3. Plant virus vectors used for expression of recombinant proteins. (A) Full virus vectors: tobacco mosaic virus (TMV, Pogue et al. 2010); cowpea mosaic virus (CPMV, Gopinath et al. 2000); potato virus X (PVX, Baulcombe et al. 1995). (B) Deconstructed virus vectors: TMV/turnip vein-clearing virus (TVCV) (Marillonnet et al. 2005); TMV (Lindbo 2007); CPMV (Cañizares et al. 2006); cucumber mosaic virus (CMV, Fukuzawa et al. 2018); bean yellow dwarf virus (BeYDV, Diamos et al. 2020). *CMV RNA1 is supplied from transgene integrated into the host genome. (C) Transgenic plants harboring viral replicons: TMV/TVCV (Werner et al. 2011), tobacco yellow dwarf virus (TYDV, Dugdale et al. 2013). Abbreviations: GOI, gene-of-interest; MP, movement protein; CP, coat protein; TGB1–3, triple gene block 1–3; Co-Pro, proteinase co-factor; NTB, NTP-binding proteins; VPg, viral protein genome-linked; Pro, proteinase; LCP, SCP, large coat protein, small coat protein; LIR, long intergenic region; SIR, short intergenic region; AlcR, alcohol receptor gene; T7p, T7 bacteriophage RNA promoter; 35Sp, cauliflower mosaic virus 35S promoter; Act2p, *A. thaliana* ACT2 promoter; AlcAp, AlcA promoter from *Aspergillus nidulans*; *PsaK2T* 5′, truncated 5′ UTR of *N. benthamiana* psaK gene; Ext 3′, intronless tobacco extensin terminator; NbACT 3′, 3′ UTR of *N. benthamiana ACT3* gene; Rb7 MAR, tobacco Rb7 matrix attachment region; NOSt, terminator of the agrobacterium nopaline synthase gene; OCSt, terminator of the agrobacterium octopine synthase gene; CaMVt, cauliflower mosaic virus terminator. The length shown in the diagram does not reflect the size of each element in virus vectors.

Full virus vectors can be used in a broad range of plant species, and appropriate virus vectors are selected depending on the plant of interest. The virus vector is introduced into plant cells by a mechanical method such as rub-inoculation (e.g., by rubbing with an abrasive) or biolistic delivery (e.g., by gene gun). Using these methods, viral RNAs, plasmid DNA carrying the viral cDNA for RNA viruses, or viral DNA for DNA viruses, are introduced into the plant tissue. Viral RNAs for RNA virus vectors are prepared by in vitro transcription from plasmids harboring viral sequences with bacteriophage promoters, such as T7 and SP6 promoters, at the 5′ end. Alternatively, viral RNAs are transcribed from DNA containing viral cDNA sequences under the control of functional promoters such as the CaMV 35S promoter within plant cells. In addition to mechanical inoculation, *Agrobacterium*-mediated delivery such as agroinfiltration using a syringe or under vacuum (agroinfection) is used to inoculate many plant RNA and DNA virus vectors into plants. Plant virus vectors based on tobacco mosaic virus (TMV), potato virus X (PVX), and cowpea mosaic virus (CPMV) have been widely used for the expression of recombinant proteins (Lico et al. 2008; Loh et al. 2017) (Table 2 and Figure 3A). The GENEWARE system, which is based on TMV, has the GOI placed between the movement protein (MP) and coat protein (CP) genes in the viral genome (Pogue et al. 2010). The vector is inoculated into plants by mechanical inoculation of infectious RNAs transcribed under the control of the T7 promoter, resulting in systemic infection and production of the recombinant proteins in plants belonging to the genus *Nicotiana*, including *N. tabacum* and *N. benthamiana*.

**Table 2:** Table 2. Plant virus vectors used for expression of recombinant proteins.

Expression system	Virus vector*	Virus genus	Virus type	Vector diagram	References
Transient expression					
Full vectors	TMV	*Tobamovirus*	RNA virus	Fig1a	Pogue et al. 2010
	PVX	*Potexvirus*	RNA virus	Fig1a	Baulcombe et al. 1995
	CPMV	*Comovirus*	RNA virus	Fig1a	Gopinath et al. 2000
Deconstructed vectors	TMV, TVCV	*Tobamovirus*	RNA virus	Fig1b	Marillonnet et al. 2005
	TMV	*Tobamovirus*	RNA virus	Fig1b	Lindbo 2007
	CPMV	*Comovirus*	RNA virus	Fig1b	Cañizares et al. 2006
	PVX	*Potexvirus*	RNA virus	—	Komarova et al. 2006
	AltMV	*Potexvirus*	RNA virus	—	Putlyaev et al. 2015
	FoMV	*Potexvirus*	RNA virus	—	Liu and Kearney 2010
	CMV	*Cucumovirus*	RNA virus	Fig1b	Fukuzawa et al. 2018
	BeYMV	*Mastrevirus*	DNA virus	Fig1b	Mor et al. 2003; Chen et al. 2013
Transgenic plant					
Deconstructed vectors	TMV, TVCV	*Tobamovirus*	RNA virus	Fig1c	Werner et al. 2011
	TYDV	*Mastrevirus*	DNA virus	Fig1c	Dugdale et al. 2013

*Abbreviations of virus name is reffered to the legend of Figure 3.

Although systemic infection and applicability in a broad range of host plants are attractive features of full plant virus vectors, one of the drawbacks is that the introduced gene fragments may be lost through deletion or mutation due to an instability of the inserts. Avesani et al. (2007) reported the stability of inserts is reversely correlated with the length in case of PVX, however, it could be affected by other factors as well such as the position of the inserts in the viral genome, and the fitness costs of inserted sequences during virus replication and systemic spread (Willemsen and Zwart 2019). This can lead to a low yield of full-length target proteins. To overcome this, and with biosafety concerns (described later), deconstructed vectors have been developed (Table 2 and Figure 3B). Some examples of deconstructed vectors include magnICON, TRBO (genus *Tobamovirus*), pEAQ (*Comovirus*), *Potexvirus*, NoPaCS (*Cucumovirus*) (all of which are RNA viruses), and *Mastrevirus* (DNA virus) (Cañizares et al. 2006; Fukuzawa et al. 2018; Giritch et al. 2006; Komarova et al. 2006; Lindbo 2007; Marillonnet et al. 2005; Mor et al. 2003). One of the most widely used systems is magnICON, which is based on TMV and turnip vein-clearing virus, and introduces viral replicons into the host plant via *Agrobacterium*. This deconstructed vector lacks the CP gene, resulting in a defect in systemic movement (Marillonnet et al. 2004, 2005). The magnICON system is able to produce GFP at up to 4 mg g^−1^ fresh weight (FW) in *N. benthamiana* (Marillonnet et al. 2005). The TRBO system uses *Agrobacterium*-mediated delivery of the TMV-based vector lacking CP genes, and can produce GFP at 3.3–5.5 mg g^−1^ FW in *N. benthamiana* (Lindbo 2007). The pEAQ vector is based on CPMV (genus *Comovirus*). The original pEAQ vector used replication-competent CPMV with the MP and CP genes replaced by the GOI in RNA2 (Cañizares et al. 2006). However, more recent versions such as pEAQ-HT use modified 5′ untranslated region (UTR) and 3′ UTR sequences of CPMV RNA2, whose replication is not supported by RNA1. Thus, the new versions of this vector do not require RNA1, indicating that they are simply overexpression vectors rather than deconstructed viral vectors (Sainsbury and Lomonossoff, 2008; Sainsbury et al. 2009). pHREAC is a recently developed CPMV-based vector with synthetic 5′ UTR and 3′ UTR sequences of CPMV. This vector is able to produce GFP at approximately 3 mg g^−1^ FW (Peyret et al. 2019). Examples of virus vectors belonging to the genus *Potexvirus* include PVX, alternanthera mosaic virus (AltMV), and foxtail mosaic virus (FoMV). In one deconstructed vector based on PVX, the CP gene and triple gene block (TGB) are replaced by GFP (Komarova et al. 2006). Another group developed a deconstructed PVX vector and a deconstructed TMV-based vector to express the light and heavy chains of antibodies within the same cells (Giritch et al. 2006). The FECT vector system was developed based on FoMV and lacks *CP* and TGB genes. FECT40, which consists of the TGB1 subgenomic RNA1 promoter comprising a putative leader sequence followed by 40 nucleotides of the TGB1 open reading frame sequence (with the AUG of TGB1 mutated to AUC), is able to produce GFP at 1.6–1.7 mg g^−1^ FW, a level similar to that obtained using the TMV-based TRBO system (described above) in the same experimental conditions in *N. benthamiana* (Liu and Kearney 2010). A deconstructed AltMV vector was developed that consisted of the TGB and another unique feature, that is, the CP gene under the control of double subgenomic promoters (which regulate the production of subgenomic RNA from a genomic RNA containing more than one open reading frame (ORF), see Newburn and White 2015) 1 and 3. This vector was found to significantly increase the production of CP (but not GFP) to 5.5 mg g^−1^ FW (Putlyaev et al. 2015). In the deconstructed AltMV vector, the CP gene can be replaced with the GOI for the expression of a recombinant protein. The NoPaCS system is based on CMV and lacks the CP gene (Fukuzawa et al. 2018). In the NoPaCS system, the CP gene in CMV RNA3 is replaced by the GOI, and the vector is agroinfiltrated into transgenic *N. benthamiana* expressing RNA1. When the GOI is the *GFP* gene, the NoPaCs system produces GFP at 0.75 mg g^−1^ FW.

For DNA virus-based systems, Geminivirus vectors are widely used. One of the most famous vectors is based on BeYDV (genus *Mastrevirus*). Many studies have focused on optimizing the components of gene expression cassettes, including the promoter, 5′ and 3′ UTR sequences, and the terminator to maximize the expression of the GOI from conventional gene expression vectors that do not use virus replicons. One of the BeYDV-based systems, pBYKEAM, can produce GFP at 3–5 mg g^−1^ FW. This system consists of the CaMV 35S promoter with a duplicated enhancer region, the truncated 5′ UTR of the *N. benthamiana* *psaK* gene, an intronless tobacco extensin terminator, the 3′ UTR of the *N. benthamiana* *ACT3* gene, and the tobacco Rb7 matrix attachment region (Diamos et al. 2016, 2020). Another system based on BeYDV, the Tsukuba system, produces GFP to at least 3.7 mg g^−1^ FW; this system consists of the CaMV 35S promoter with a duplicated enhancer region, the 5′ UTR of the *Arabidopsis thaliana* alcohol dehydrogenase gene (Sugio et al. 2008), the terminator of a heat shock protein gene (Nagaya et al. 2010), and the tobacco extension gene 3′ element with simultaneous expression of the p19 gene from tobacco bushy stunt virus (Yamamoto et al. 2018). The pRIC system, which is also based on BeYDV, uses the tobacco Rb7 matrix attachment region (Regnard et al. 2010).

Stable transgenic plants expressing viral replicons are difficult to generate because virus replication could negatively affect host cell viability, but there are a few successful examples (Table 2 and Figure 3C). Stable transgenic *N. benthamiana* plants expressing a TMV/turnip vein-clearing virus (TVCV)-based vector lacking the CP gene, as used in the magnICON system, were not obtained, even when the expression of replicons was regulated by an ethanol-inducible promoter. This was probably because of background leaked release of viral replicons (Werner et al. 2011). This obstacle was countered by removing the MP (which functions in virus cell-to-cell movement and would not be required in transgenic plants because virus replication occurs every transformed cell) from the inducible viral replicons to avoid the virus spreading from cells where it sporadically replicates during transformation. Regardless of the non-essential gene, the supply of MP regulated by the ethanol-inducible promoter resulted in a higher level of GFP production (2.7–4.3 mg g^−1^ FW), a comparable level to that achieved by transient expression by agroinfiltration (Marillonnet et al. 2005; Werner et al. 2011). In the INTACT system using a Geminivirus vector based on tobacco yellow dwarf virus, transgenic plants are able to produce viral replicon elements containing the GOI under the control of the CaMV 35S promoter, and the *Rep*/*RepA* gene, which is required for replication of viral replicons, under the control of the ethanol-inducible promoter. One of the unique features is that the GOI is split into two exons by an intron, and the full-length mRNA is generated only after the circularization of viral DNA in the presence of Rep/RepA and removal of the intron (Dugdale et al. 2013).

In the studies discussed in this section, GFP yield was used as the indicator of vector performance. However, simple comparisons should be made with caution because protein yield strongly depends on the experimental conditions (e.g. growth temperature, humidity, and light intensity) (Fujiuchi et al. 2016). After introducing deconstructed virus vectors into plants by *Agrobacterium*, the efficiency of protein expression depends on the infection and gene-delivery efficiencies of *Agrobacterium* in the host (Liu and Kearney 2010; Peyret and Lomonossoff 2015).

We should also consider the containment method when using engineered virus vectors (and when using other agents including *Agrobacterium*) to address biosafety concerns (Brewer et al. 2018; Pasin et al. 2019). Compared with full virus vectors, the deconstructed vectors described above have a significantly lower risk of spreading the virus in nature because they lack gene(s) for systemic infection. The CMV-based deconstructed vector system retains systemic infection ability, but only in helper plants expressing the MP. Therefore, the virus can be successfully contained (Fukuzawa et al. 2011). The CMV vectors lacking the MP encoded in RNA3 do not infect wild-type plants because of the defect in cell-to-cell movement, but they are able to infect systemically and express the GOI in helper transgenic plants expressing the CMV MP gene. In this system, progeny viruses collected from the helper transgenic plants do not systemically infect wild-type plants. Furthermore, no recombination occurs between viral RNA3 (which has a stop codon slightly downstream of the start codon of the MP gene) and the transgene MP transcripts. Thus, the CMV-vector system successfully reduces the risk of the virus spreading in nature.

## Host engineering for higher protein expression

Various plant species have been used to express recombinant proteins through stable expression systems. However, among the transient expression systems, *N. benthamiana*, a close relative of *N. tabacum*, is almost exclusively used because of its specialized nature to allow high protein expression of foreign gene, ease of cultivation and agroinfiltration, good size, high growth speed, and simple genome structure. *N. benthamiana* is a widely used model plant in studies on responses to viruses and other diseases because of its unique susceptibility to diverse plant pathogens (Bally et al. 2018). In general, if a foreign gene is highly expressed in plants, the gene silencing mechanism is activated. This degrades transcripts of the introduced gene and represses its transcription, resulting in decreased expression of the target protein. In plant virus-based RNA expression systems, the post-transcriptional gene (RNA) silencing mechanism (PTGS) of the plant acts against the introduced virus in plants by specifically degrading the viral genome. To counter this, plant viruses encode viral RNA silencing suppressors (VRS) in their genomes that inhibit the PTGS (Li and Wang 2019). A *N. benthamiana* line permanently expressing these VRS was created (Fukuzawa et al. 2010a). To suppress the cyclic symptoms in which the expression level caused by the CMV vector varied among different leaves of the plant, a transgenic line harboring a potyvirus helper component-proteinase (HC-Pro), one of the VRS, was created. This plant constitutively expresses HC-Pro silencing suppressor derived from another plant virus, allowing for a high expression level of the GOI. The suppression of the PTGS using these VRS-expressing recombinant *N. benthamiana* lines is a good strategy to enhance the expression of foreign genes.

During RNA silencing, first, double-stranded RNA (dsRNA) is synthesized by RNA-dependent RNA polymerases (RDR) using aberrant single-stranded RNAs, such as viral RNAs and mRNAs, as templates. Dicer-like proteins (DCL) cleave the dsRNAs to generate small interfering RNAs or micro interfering RNAs. These small RNAs are captured by Argonaute proteins (AGO) and then incorporated into RNA-induced silencing complexes for induction of sequence-specific RNA degradation or inhibition of translation (Csorba et al. 2009). In this way, repression of the RNA silencing mechanism can increase recombinant protein production. There are several reports on the repression or knockout of RNA silencing-related genes in *N. benthamiana*.

An AGO1-knockout *N. benthamiana* line was produced by clustered regularly interspaced palindromic repeats/CRISPR-associated protein 9 (CRISPR/Cas9) genome-editing technology (Ludman and Fátyol 2021). Studies on this line indicated that protection against wild-type turnip crinkle virus was dependent on AGO1. AGO2-knockout *N. benthamiana* was also produced using CRISPR/Cas9 technology, and it exhibited differential sensitivities towards various viruses (Ludman et al. 2017; Kenesi et al. 2021). AGO5-knockout *N. benthamiana* plants (*nbago5* plants) were produced using CRISPR/Cas9 technology as well (Tu et al. 2023). Analyses of those plants revealed that NbAGO5 provides defense against bamboo mosaic virus, PVX, TMV, and a mutant CMV deficient in the *2b* gene, but not against the wild-type CMV and turnip mosaic virus (Tu et al. 2023). These studies suggest that suppression of AGO1, AGO2, or AGO5 may increase virus accumulation leading to higher amount of recombinant protein production. Conversely, a study in which the *AGO10* gene of *N. benthamiana* was repressed by virus-induced gene silencing revealed that NbAGO10 positively regulates bamboo mosaic virus accumulation (Huang et al. 2019). The authors of that study concluded that it may be more advantageous for recombinant protein production if NbAGO10 is not repressed.

Two different groups produced *RDR6*-knockout lines of *N. benthamiana* almost simultaneously (Ludman and Fátyol 2019; Matsuo and Atsumi 2019). A transient *GFP* gene expression assay showed that, compared with wild-type plants, the RDR6-knockout *N. benthamiana* plants (*rdr6* plants) produced about 2.5- and 2.2-fold higher levels of GFP and its mRNA (Matsuo and Atsumi 2019).

*DCL2* and *DCL4* genes were repressed singly and in combination in *N. benthamiana* by RNA-interference (RNAi) technology (ΔD2, ΔD4, and ΔD2ΔD4 plants) (Matsuo and Matsumura 2017). The levels of transiently expressed GFP and human fibroblast growth factor 1 mRNAs were about 2-fold and 5-fold higher, respectively, in ΔD2ΔD4 plants than in ΔD2, ΔD4, and wild-type plants. Thus, it was clear that the *DCL2* and *DCL4* genes must be repressed simultaneously to increase the production of the recombinant protein in plants. *N. benthamiana* plants with simultaneous knockout of *DCL2* and *DCL4* were also produced (*dcl2 dcl4* plants) using CRISPR/Cas9 technology (Matsuo 2022). Compared with wild-type and *rdr6* plants, the *dcl2 dcl4* plants produced more human fibroblast growth factor 1.

Although the production of recombinant proteins in other *N. benthamiana* mutants with repressed or knocked-out RNA silencing-related genes has not been investigated, the fact that the amount of virus-derived RNA was increased in the mutants discussed above suggests that other mutants may also be suitable for the production of recombinant proteins.

## Host engineering for modification of glycans

Glycosylation is an important post-translational modification in plant and mammal cells. Glycans contribute to protein function and stability and play an important role in the quality of recombinant proteins. There are two main types of glycans that modify proteins: *N*-linked glycans that bind to asparagine residues (Asn); and *O*-linked glycans that bind to serine/threonine residues (Ser/Thr). Because there are structural differences between plant-derived and mammal-derived glycans, many studies have attempted to convert the structure of plant-derived glycans to the mammal type.

### 1) Deletion of plant-specific sugar-residues from plant *N*-glycans

**Figure 4:**
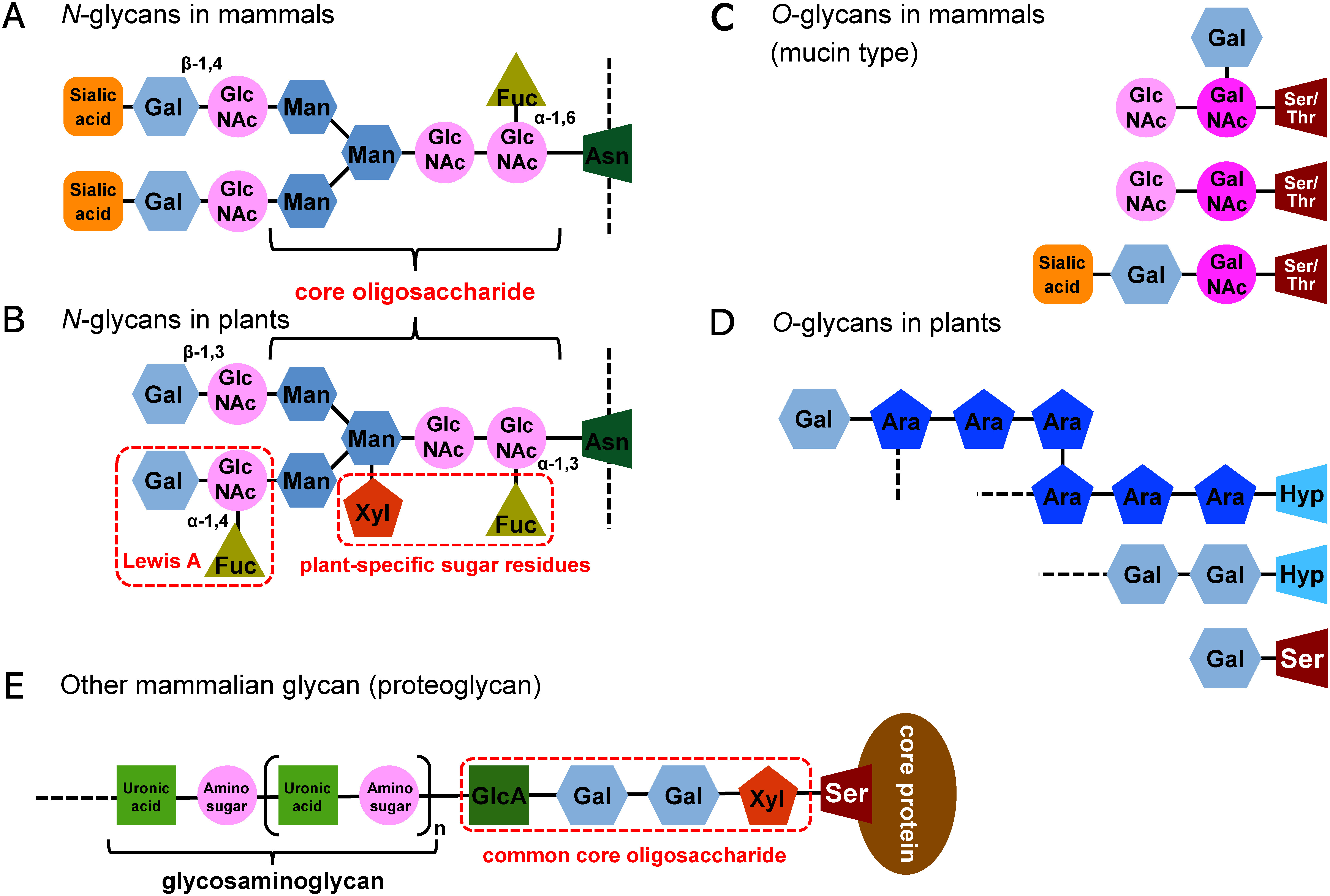
Figure 4. Variety of glycans in mammals and plants. (A and B) Plant *N*-glycans mainly differ from mammalian *N*-glycans by the followings: absence of sialic acid, presence of α(1,3) linked fucose residues on the reducing terminal GlcNAc instead of α(1,6)-linked fucose residues in mammalian *N*-glycans, and presence of β(1,2) linked xylose residues on the mannose residues of the core oligosaccharide, common moiety between plant and mammalian *N*-glycans. The α(1,3) linked fucose and β(1,2) linked xylose residues are also known as the plant-specific sugar residues. Interestingly, plant *N*-glycans frequently have Lewis A antigen, one of human blood group, at their non-reducing end. (C and D) In many cases, *N*-acetylgalactosamine is firstly linked to serine/threonine residues of proteins in mammalian *O*-glycans. On the other hand, in plant *O*-glycans, arabinose and galactose are linked to hydroxyproline and serine residues of proteins. Sialic acid is also absence for plant *O*-glycans. (E) Glycosaminoglycans are the major mammalian sugar chains and known as sugar parts of proteoglycans. Glycosaminoglycans are a group of anionic polysaccharides composed of sulfated repeating disaccharide building blocks, consist of uronic acids and amino sugars, and are synthesized through common core oligosaccharides on serine residues of core. Glycosaminoglycans include such as heparin, heparan sulfate, and keratan sulfate. Only major glycans are shown in this figure, and glycans show further diversity in various species. Abbreviations: Ara, arabinose; Asn, asparagine; Fuc, fucose; GalNAc, *N*-acetylgalactosamine; GlcA, glucuronic acid; GlcNAc, *N*-acetylglucosamine; Gal, galactose; Hyp, hydroxyproline; Man, mannose; Ser, serine; Thr, threonine; Xyl, xylose.

*N*-glycans of plants and mammals have a common core oligosaccharide structure (Man3GlcNAc2) that is covalently linked to Asn residues in the consensus sequence (Asn-X-Ser/Thr) of proteins (Figure 4A, B). There are several differences between plant and mammal *N*-glycans (Balen and Krsnik-Rasol 2007; Gomord et al. 2010). Plant *N*-glycans do not have sialic acid residues and core α-1,6-fucose residues, but instead have β-1,2-xylose and/or α-1,3-fucose residues, known as plant-specific sugar residues, linked to the *N*-acetylglucosamine residues of their core oligosaccharide (Figure 4B). Plant-produced glycoproteins are immunogenic because of the presence of these plant-specific sugar residues in *N*-glycans (van Ree et al. 2000; Wilson et al. 1998). It has been reported that the sera of approximately 50% of non-allergic people contain β-1,2-xylose-specific antibodies, and the sera of approximately 25% of non-allergic people contain α-1,3-fucose-specific antibodies (Bardor et al. 2003). Furthermore, the trisaccharide Galβ1-3[Fucα1-4]-GlcNAc, known as the Lewis A antigen, forms at the non-reducing terminal of plant *N*-glycans (Figure 4B). Thus, to change plant *N*-glycans to mammal type, β-1,4-galactosyltransferase must be expressed in plants instead of β-1,3-galactosyltransferase.

Plant-specific sugar residues can be deleted from *N*-glycans by stopping *N*-glycan elongation at the early stages of biosynthesis. For example, a recombinant KDEL (an endoplasmic reticulum retention peptide sequence)-tagged protein was produced in transgenic rice seeds and possessed a high-mannose type *N*-glycan lacking β-1,2-xylose or α-1,3-fucose residues (Wang et al. 2013). When *N. benthamiana* grown in hydroponic culture was treated with kifunensin, an α-mannosidase I inhibitor, the *N*-glycans on the recombinant proteins became mostly the Man9 high-mannose type without plant-specific sugar residues (Roychowdhury et al. 2018).

The β-1,2-xylose and α-1,3-fucose residues are transferred from UDP-xylose and GDP-fucose to the common core oligosaccharide by β-1,2-xylosyltransferase and α-1,3-fucosyltransferase, respectively (Schoberer and Strasser 2018). Therefore, repression or knockout of the respective transferase genes has been performed to remove the plant-specific sugar residues in species such as *A. thaliana* (Schähs et al. 2007; Strasser et al. 2004) and other plants using RNA interference (RNAi) technology, the CRISPR/Cas9 system, or other systems. In *N. benthamiana*, six glycosyltransferase genes related to the formation of plant-specific sugar residues were edited by CRISPR/Cas9 technology (FX-KO plants) (Jansing et al. 2019). The recombinant 2G12 antibody transiently expressed in the FX-KO plants contained *N*-glycans completely lacking plant-specific sugar residues (Jansing et al. 2019). The use of virus-induced gene silencing and RNAi technologies to repress the expression of the gene encoding GDP-D-mannose 4,6-dehydratase, which is related to GDP-fucose synthesis, also led to the deletion of α-1,3-fucose residues from the common core oligosaccharide (Matsuo and Matsumura 2011; Matsuo et al. 2014).

### 2) Addition of sialic acid to plant *N*-glycans

It has been reported that there is a relationship between the amount of sialic acid and in vivo protein stability, and thus many studies have attempted to add sialic acid to plant-produced glycans (Schoberer and Strasser 2018). Because plants lack the sialic acid synthesis system, it is necessary to introduce essential components of this system into plants to add sialic acid to plant-produced glycans. In addition to the genes required for sialic acid biosynthesis itself, genes encoding β-1,4-galactosyltransferase, sialic acid transporters, and sialyltransferases are essential for the construction of sialylated *N*-glycans in plants. A multi-α-2,6-sialylated recombinant human erythropoietin fusion protein was successfully produced by transiently expressing 11 human enzymes acting in different subcellular compartments at different stages of the glycosylation pathway in *N. benthamiana* plants with repressed β-1,2-xylosyltransferase and α-1,3-fucosyltransferase (ΔXTFT^Sia^ plants) (Castilho et al. 2013). Recombinant α1-antitrypsin, erythropoietin, and IgG with α2,6- or α2,3-sialylated *N*-glycans were also produced in ΔXTFT^Sia^ plants (Kallolimath et al. 2016). Furthermore, the polysialylated (degree of polymerization >40) Ig5FN1 module of neural cell adhesion molecule 1 was also produced in ΔXTFT^Sia^ plants by transiently expressing human α-2,8-polysialyltransferases (Kallolimath et al. 2016). The plant-made polysialic acid was confirmed to be functionally active in cell-based inhibition assays.

### 3) Construction of mammal type *O*-glycans in plants

The structure of *O*-glycosylation also differs between plants and mammals. The most common *O*-glycosylation in mammal cells is *N*-acetylgalactosamine (GalNAc) substitution on Ser/Thr residues of proteins (Figure 4C); whereas the predominant *O*-glycan in plant cells is galactose or arabinose substitution on Ser or hydroxylated proline residues of proteins (Figure 4D; Saito et al. 2014; Showalter and Basu 2016). Therefore, to achieve mammalian-type glycosylation in plants, it is necessary to construct the mammalian-type *O*-glycosylation pathway (Strasser 2012). To date, this has been achieved in plants by introducing various mammal-derived glycosyltransferases (Castilho et al. 2012; Daskalova et al. 2010; Dicker et al. 2016). Successful sialylated mammalian-type *O*-glycosylation has also been achieved in *N. benthamiana* (Castilho et al. 2012). To prevent undesired plant-type *O*-glycosylation of recombinant proteins, the activity of proline hydroxylase must be eliminated. Several studies have accomplished this in a range of different plants, and in all cases, proline hydroxylation of the recombinant protein was inhibited (Parsons et al. 2013).

### 4) Construction of other mammal-type glycans in plants

Proteoglycans are major glycoproteins that are widely distributed in mammals, insects, and fish (Lindahl et al. 2017). Proteoglycans consist of a core protein and a glycan moiety (Figure 4E). The glycan portion further consists of a core oligosaccharide and glycosaminoglycans (Figure 4E). Glycosaminoglycans can be classified as hyaluronan, chondroitin sulfate, dermatan sulfate, heparin, heparan sulfate, or keratan sulfate according to their structure and the ratio of sulfation. The different glycosaminoglycans confer various biological functions upon proteoglycans, including involvement in the extracellular matrix, cell to cell interactions, and cell growth. Glycosaminoglycans are covalently attached to a Ser residue in the core protein by the common tetrasaccharide glucuronic acid-β-1,3-galactose-β-1,3-galactose-β-1,4-xylose, namely, the core oligosaccharide (Figure 4E; Lindahl et al. 2017). Thus, *O*-xylation of Ser is the initial step of glycosaminoglycan biosynthesis, and is catalyzed by xylosyltransferases 1 and 2 (XYLT1 and XYLT2) (Pönighaus et al. 2007; Schön et al. 2006). Transiently expressed serglycin, one of the core proteins of proteoglycan, was successfully xylosylated by human XYLT2 co-expressed in *N. benthamiana* (Matsuo and Atsumi 2018). These results indicate that glycosaminoglycans may be synthesized successfully in plants in the future.

## Plant cultivation

When cultivating plants to produce PMP, physical containment facilities are required to prevent the spread of GM organisms to the outside. In addition, a cultivation environment under which plants can be stably cultivated without being affected by climate change and/or pests is required. Greenhouse- or vertical farming-type operations are suitable as cultivation facilities, and open field cultivation should be avoided (Huebbers and Buyel 2021). Traditionally, *N. benthamiana* is cultivated in soil. However, in large-scale production facilities for PMP, hydroponic cultivation has been adopted in addition to soil cultivation. For example, KBio (formerly KBP) Inc., which produces cytokines, enzymes, antibodies, and vaccines, cultivates *N. benthamiana* in soil in a greenhouse with light supplied by metal halide lamps. The facility is equipped with a semi-automatic agroinfiltration device (Pogue et al. 2010). Aramis Biotechnologies (formerly Medicago) Inc. cultivate *N. benthamiana* in soil in a greenhouse, and their production plant is equipped with automated infection, extraction, and purification facilities according to publicly available videos (https://www.youtube.com/watch?v=8cGnqq0ayls). iBio (formerly Caliber Biotherapeutics) Inc. has a large-scale cultivation facility with a fully artificial light system. *N. benthamiana* is cultivated using a vertical farming method with a rock wool hydroponic system, under light supplied by light-emitting diodes. The facility is equipped with an agroinfiltration device and extraction and purification facilities, and their system can process more than 3,500 kg plant biomass per week (Holtz et al. 2015).

Hydroponic cultivation is a method of growing plants by immersing them in a nutrient solution, without soil. Because the nutrients and water can be directly absorbed, this system promotes plant growth, and it has a small environmental impact with an excellent life cycle assessment (Wimmerova et al. 2022). In addition, when used for transient expression of PMP, there is the advantage that inactivation of the soil after agroinfiltration is not necessary. It has been reported that the intracellular concentrations of antioxidants, namely ascorbic acid and tocopherol, are increased hydroponically grown plants (Buchanan and Omaye 2013). Ascorbic acid removes reactive oxygen species during *A. tumefaciens* infection, suppresses necrosis, and induces higher accumulation of target proteins (Nosaki et al. 2021). Therefore, it is considered to be a significant advantage to use hydroponically cultivated plants for transient expression of proteins for PMP.

Several studies have compared PMP production between soil cultivation and hydroponic cultivation systems. Frigerio et al. (2022) used agroinfiltration to produce *N. benthamiana* transiently expressing the SARS-CoV-2 antibody, and compared plants grown under hydroponic cultivation using rock wool with those grown in soil. There was no significant difference in antibody production and antigen neutralization activity between the two plant materials. Nguyen et al. (2023) produced *N. benthamiana* transiently expressing Varlilumab (a monoclonal antibody against CD27) by the agroinfiltration method, and compared plants grown using the nutrient film technique of hydroponic cultivation with those grown in soil. They found that antibody production was 3.5-times higher in the hydroponically cultivated plants than in the soil-cultivated plants. Furthermore, they reported that the glycosylation characteristics of the antibody protein differed between the two materials. The nutrient film technique is a hydroponic cultivation method that does not use a supporting material, and is superior to the rock wool method in terms of nutrient and oxygen supply (Huebbers and Buyel 2021). This difference is thought to affect plant physiology. As a result, it has been suggested that different cultivation methods may affect the volume and quality of the target protein.

Seasonal changes can also affect yield and quality of recombinant proteins produced in GM plants (Knödler et al. 2019). Therefore, it is desirable to cultivate plants in closed facilities equipped with artificial lighting for the production of PMP. Studies have reported that the amount of recombinant protein can be increased by reducing the cultivation temperature after agroinfiltration (Matsuda et al. 2017) and by increasing the nitrogen concentration in the hydroponic nutrient solution (Fujiuchi et al. 2016). In the future, further adjustments of the cultivation environment are expected to increase PMP production.

## Future perspectives

As described and discussed in this review, research on PMP has a long history. The trend has gradually shifted from production in stably transformed transgenic plants to transient production in *N. benthamiana*. This is because of the many advantages of the latter system, namely *N. benthamiana*’s ability to express one or more foreign genes quickly and in large amounts, its ease of cultivation and fast growth, the availability of multiple expression systems and experimental procedures that can be automated, and the array of engineered host strains. However, there are still some reservations: it accumulates tobacco alkaloids as well as abundant Rubisco protein in the leaves, there is a requirement for transformation each time, the product must be purified after being produced, and some consumers still have a negative view of products derived from “tobacco” even though there are many positive opinions (Milne 2008; Nevitt et al. 2006). If the intention is to produce edible pharmaceuticals, other hosts such as strawberry, soybean, or potato should be selected. Thus, there is scope for improvement of systems using crop species as the hosts for PMP production. Edible pharmaceuticals such as edible vaccines or similar products could be next-generation PMP. The strawberry line expressing canine interferon-α introduced in this review is a good example. Another example is rice plants expressing the Japanese cedar pollen allergen in the grains, which was shown to alleviate hay fever symptoms by long-term intake (Endo et al. 2021; Hashimoto et al. 2022). Because cold-storage supply chains are not well established in some developing countries, edible vaccines could be a good solution for responding to outbreaks of new infectious diseases in the future.

Considering the small environmental impact of PMP, this system should be more widely adopted. However, the need to cultivate transgenic plants in an enclosed environment, such as a plant factory, increases the initial investment costs, operation costs, and life cycle assessment outcome in terms of greenhouse gas emissions. There is a need to increase public acceptance of the cultivation of transgenic plants in open fields. In addition, it will be important to develop next-generation low-cost high-efficient plant factories that use sunlight, thermal energy, and/or heat and CO_2_ outputs from industrial factories or power plants, and upcycle unused plant residues such as stems and roots for the production of biofuels and other materials. Even after such upcycling, non-negligible wastes will be produced by plant factories, and their bioremediation by microorganisms and other organisms including insects is another important issue. The use of beneficial microorganisms for plant growth could be another area of development, given it is more practical to apply biostimulants in enclosed environments than in open fields.

As discussed in this review, further research on PMP not only includes the development and refinement of pharmaceutical production methods but also the development of next-generation plant cultivation systems. Such systems could be even applied to studies on space exploration and immigration. We believe that continuous efforts in this area will provide alternative choices for pharmaceutical production in plants in an eco-friendly manner, with potential applications in many other fields related to plant cultivation, growth-promoting microorganisms, and waste recycling.

## Abbreviations

Ago: Argonaute protein
AltMV: alternanthera mosaic virus
BeYDV: bean yellow dwarf virus
CaMV: cauliflower mosaic virus
CMV: cucumber mosaic virus
CP: capsid/coat protein
CPMV: cowpea mosaic virus
CRISPR/Cas9: clustered regularly interspaced palindromic repeats/CRISPR-associated protein 9
DCL: dicer-like protein
FDA: Food and Drug Administration
FoMV: foxtail mosaic virus
GFP: green fluorescent protein
GM: genetically modified
GMP: good manufacturing practice
GOI: gene of interest
MP: movement protein
ORF: open reading frame
PMF: plant molecular farming
PMP: plant-made pharmaceuticals
PTGS: post-transcriptional gene silencing
PVX: potato virus X
RDR: RNA-dependent RNA polymerases
TGB: triple gene block
TMV: tobacco mosaic virus
UTR: untranslated region
VRS: viral RNA silencing suppressors

## Description of Supplementary Files

Supplementary Table S1 List of studies focusing on plant-made COVID-19 vaccine/pharmaceutical
